# Espressogate

**DOI:** 10.1038/s44319-024-00247-x

**Published:** 2024-09-16

**Authors:** David R Smith

**Affiliations:** Western University, London, ON N6A 3K7 Canada

**Keywords:** Economics, Law & Politics, History & Philosophy of Science

## Abstract

A food services scandal as a sign of bigger problems that haunt universities.

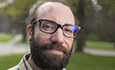

*“Even bad coffee is better than no coffee at all.” – David Lynch*

Hard thinking requires strong stimulants. For my scientific sustenance, I rely on the double espresso. Without my caffeine, I’m useless as a scientist, teacher and leader of a research group. I limit myself to two double shots a day, one at breakfast and another after lunch. When I’m on campus, I get my espresso at a small take-away sandwich spot called Einstein’s Café.

I love Einstein’s. It’s the perfect distance from my office to stretch the legs and reset the mind. I can also access it from an underground tunnel when the weather is bad. I’ve been going there almost daily for more than 10 years. I’m a familiar customer now and the staff start getting my espresso ready as soon as I walk through the door.

I have my order down to an art. Einstein’s only has disposable cups, but at least they are compostable. I always take my double espresso in their macchiato cup, which is larger than the tiny espresso cup. This is because the espresso cups don’t come with lids and are too small to add further liquids. Bear with me through this minutia as it is crucial to the story.

Once I’m handed my espresso in the macchiato cup with a lid, I walk over to a customer-facing hot water dispenser used mostly by tea drinkers and add about a tablespoon of near-boiling water. I typically like espresso with longer pull times – long shots – but Einstein’s automated espresso machine can’t do this, so I cheat by adding a bit of hot water. Finally, I go to the dairy bar and add a teaspoon of cream. Voilà, I now have the perfect fuel to sustain hours of intense science.

The other day I popped into Einstein’s for my afternoon fix and the staff looked at me strangely. There was no “Hey Dave, how’s it going?” No, “Nice to see you.” Instead, they all stood back and let the manager come forward. She asked me what I would like. I said, “A double espresso please.” A few moments later she placed the drink on the counter. To my astonishment, it was in a tiny cup without a lid. “No worries,” I said, “but I usually like my double shot in the slightly larger macchiato cup with a lid.” This is when things took a turn for the worst.

“Sir, we will no longer be giving you your espresso in the macchiato cup. If you want a larger cup, you need to order an Americano.” “Alright, how much is an Americano?” “Three dollars and sixty cents.” This was sixty cents more than my double shot. She followed this by saying, “Also, sir, you can no longer add any dairy to your double espresso. If you want dairy, you need to order the Americano.” I was in shock. I grabbed my lidless, tiny-cup espresso and walked away, spilling half of it on my pants as I went.

Back in my office, I stewed things over. I’m no penny pincher, but the thought of having to pay an additional 60 cents for a tablespoon of hot water and touch of cream got under my skin. Also, at Einstein’s an Americano is normally served in a large cup (not a macchiato cup) with about 250 ml of hot water. Moreover, if customers buying a drip coffee for less than two dollars can use the dairy bar why can’t someone buying a three-dollar espresso do the same? It all felt petty and mean-spirited, especially for someone who has spent thousands of dollars at Einstein’s over more than a decade.

Once I calmed down, I went back to Einstein’s to see if I could reason with the manager. I explained how I was only adding a drop of hot water and very little cream and that I was doing this all myself, not troubling the staff. “If you must know, people have been complaining about you,” she said. “They see that you are ordering a drink that doesn’t exist.” Things got nasty when she said that it was not normal to add cream to an espresso. She grew up in Europe and had never seen such a thing. The interaction ended with her handing me the business card of an operations manager from Hospitality Services. “If you have a problem, talk to him!” she said.

I had better things to do than get into arguments about the nuances of espresso, but it was the principle of the thing that kept me going. At Western University, all the food and dining locations, including Einstein’s, are managed and operated by Hospitality Services. The person on the business card, Brady, appeared to be quite high up in the hierarchy of that division. I spent the remainder of the day writing an email to Brady, outlining my case. I even made detailed PowerPoint slides showing the different cup sizes at Einstein’s.

It took a few days for me to receive a reply. In the interim, I had told students, friends and colleagues about the Einstein’s incident, which I was calling “Espressogate”. They all got a kick out of it and were desperate to learn about the outcome. I’ll save you the suspense. In the end I was offered a $20 gift card to any eatery on campus. But if I wanted my usual drink, I had to order and pay for an Americano. Their reply contained a lot of platitudes: “We definitely want to ensure you get your espresso fix and are able to enjoy it the way you like it. The Einstein’s set coffee menu allows for some customization, but the line may get a little blurry when that customization makes the drink into something else that already exists on the menu…”

I kept the fight going for another two weeks, arguing that in the very least I should be able to order my drink in a cup with a lid on it. I also visited other coffee shops on campus, to see if they would serve me a double espresso in a macchiato cup (some did). I even spoke in-person with someone from Hospitality Services. In the end, I realized that someone in an administrative office far away from Einstein’s or lecture halls had made a bureaucratic decision about coffee drinks on campus and everyone has had to follow the memo, no questions asked. Maybe it is silly that I took espressogate so far, but I believe that this small dispute reflects a bigger problem on campus.

I’ve not been back to Einstein’s since that day. Occasionally, I trek across the university campus to the nearest Starbucks, where I can get a long espresso. But most days I take my afternoon espresso alone in my office. I’m sad to admit that at the far end of my desk there now sits a plastic Nespresso machine, just like you’d find in a corporate office.

## Supplementary information

Peer Review File

