# Supplementary material for: Espressogate
Source: EMBO Rep. 2024 Sep 16;25(10):4093–4. doi: 10.1038/s44319-024-00247-x (PMC11467293; doi:10.1038/s44319-024-00247-x)
Supplement: Supplementary file 1 — Peer Review File [file 44319_2024_247_MOESM1_ESM.pdf]

EMBO reports

Espressogate

David Smith

Corresponding author(s): David Smith (dsmit242@uwo.ca)

Review Timeline:

Submission Date:	27th Aug 24
Accepted:	28th Aug 24

Editor: Holger Breithaupt

Transaction Report: This article type is not peer reviewed.
